# PKA compartmentalization links cAMP signaling and autophagy

**DOI:** 10.1038/s41418-021-00761-8

**Published:** 2021-03-19

**Authors:** Francesca Grisan, Liliana F. Iannucci, Nicoletta C. Surdo, Andrea Gerbino, Sofia Zanin, Giulietta Di Benedetto, Tullio Pozzan, Konstantinos Lefkimmiatis

**Affiliations:** 1 Foundation for Advanced Biomedical Research, Veneto Institute of Molecular Medicine, Padua, Italy; 2 Department of Biology, University of Padua, Padua, Italy; 3 Department of Molecular Medicine, University of Pavia, Pavia, Italy; 4 Department of Biosciences, Biotechnology and Biopharmaceutics, University of Bari, Bari, Italy; 5 Neuroscience Institute, National Research Council, Padua, Italy

**Keywords:** Kinases, Autophagy

## Abstract

Autophagy is a highly regulated degradative process crucial for maintaining cell homeostasis. This important catabolic mechanism can be nonspecific, but usually occurs with fine spatial selectivity (compartmentalization), engaging only specific subcellular sites. While the molecular machines driving autophagy are well understood, the involvement of localized signaling events in this process is not well defined. Among the pathways that regulate autophagy, the cyclic AMP (cAMP)/protein kinase A (PKA) cascade can be compartmentalized in distinct functional units called microdomains. However, while it is well established that, depending on the cell type, cAMP can inhibit or promote autophagy, the role of cAMP/PKA microdomains has not been tested. Here we show not only that the effects on autophagy of the same cAMP elevation differ in different cell types, but that they depend on a highly complex sub-compartmentalization of the signaling cascade. We show in addition that, in HT-29 cells, in which autophagy is modulated by cAMP rising treatments, PKA activity is strictly regulated in space and time by phosphatases, which largely prevent the phosphorylation of soluble substrates, while membrane-bound targets are less sensitive to the action of these enzymes. Interestingly, we also found that the subcellular distribution of PKA type-II regulatory PKA subunits hinders the effect of PKA on autophagy, while displacement of type-I regulatory PKA subunits has no effect. Our data demonstrate that local PKA activity can occur independently of local cAMP concentrations and provide strong evidence for a link between localized PKA signaling events and autophagy.

## Introduction

Autophagy is a degradative process triggered by starvation to help cells compensate for the lack of nutrients [1]. Though starvation-induced autophagy is non-selective, a selective form of this process can contribute to cell homeostasis of non-starved cells by eliminating damaged or excess material [2]. Autophagy targets aggregated proteins or dysfunctional organelles that could be detrimental for the cell [3, 4]; accordingly, its deregulation leads to accumulation of damaged cellular components and it is a causal factor for several diseases including neurodegenerative disorders [5], cardiovascular diseases [6], and cancer [7, 8].

Autophagy is characterized by a chain of molecular events that ensure the recognition of damaged material (cargo), its tethering to the autophagosome, and degradation of the cargo to its constituents. Most components and effectors of autophagy are well known; nevertheless, our understanding of the signaling events governing this process is not complete [2, 9]. For instance, post-translational modifications [9, 10], such as phosphorylation and ubiquitination, are major regulators of autophagy, but the molecular mechanisms through which these processes tune autophagy to cellular metabolism are not fully elucidated. A master regulator of autophagy is the mammalian target of rapamycin (mTOR), a serine/threonine kinase with energy sensing functions [11]. mTOR interacts with regulatory-associated protein of mTOR (RAPTOR) [12] and other proteins to form mTORC1, a complex that directly suppresses autophagy in response to the nutrient status of the cells [13, 14]. Another kinase that regulates autophagy, independently from mTOR, is the cyclic AMP (cAMP)-activated protein kinase A (PKA) [15].

PKA is a tetramer composed of two regulatory (PKA-R) and two catalytic (PKA-C) subunits [16]. There are two catalytic isoforms, PKA-Cα or PKA-Cβ [16–19]. Similarly, two types of regulatory subunits have been identified, PKA-RI and PKA-RII [16], each having two isoforms, RIα, RIβ, RIIα, and RIIβ, respectively [16, 20]. Depending on the regulatory subunits, PKA is designated as PKA type I when PKA-C is complexed with PKA-RI and PKA type II when in the complex includes PKA-RII [17]. Importantly, PKA-Rs associate with a family of tethering proteins called A kinase anchoring proteins (AKAPs) to determine the subcellular localization of PKA holoenzymes [16, 21]. AKAPs act as molecular platforms where functional signalosomes called microdomains containing PKA, its substrates, and signaling molecules of the cAMP pathway (phosphatases, phosphodiesterases (PDEs)) [16, 22] are generated. The formation of cAMP microdomains requires the coordination between AKAPs and regulatory elements such as PDEs (whose hydrolyzing action confines the messenger diffusion [23]) and phosphatases [24, 25] (that restrict the actions of PKA). While the topological distribution of cAMP microdomains is crucial for the functional outcomes of the cAMP/PKA signaling axis [16, 26], identifying the link between microdomains and cellular outcomes remains a challenging task.

We discovered that cAMP increases have different effects on autophagy depending on the cellular model: in HT-29 cells cAMP elevation results in increased autophagic flux but have no effect in HeLa cells. Moreover, we demonstrate that these apparently contradictory effects depend on the different mechanisms that control cAMP/PKA compartmentalization in the two cell lines. In particular, PKA-dependent phosphorylation in HT-29 cells is high at the membranes (mitochondria, endoplasmic reticulum, and plasma membrane), but nearly absent in the soluble fractions of the cell, even at saturating cAMP levels, while in HeLa cells, PKA-dependent phosphorylation faithfully mirrors the intracellular levels of cAMP [27, 28]. We identify phosphatases as the main mechanism through which compartmentalization of PKA-dependent phosphorylation is achieved in HT-29 cells. To test a possible connection between PKA compartmentalization and the cAMP effects on autophagy, we employed genetically encoded peptides able to selectively displace PKA-I or PKA-II holoenzymes. Interestingly, we found that only displacement of PKA-II reversed the effects on autophagy, providing a link between cAMP/PKA microdomains and this process.

## Materials and methods

### Reagents

Forskolin (FSK), H-89 dihydrocloride (H89), Calyculin A (CalA), 3-isobutyl-1-methylxantine (IBMX), Chloroquine (ChQ), dimethyl sulfoxide (DMSO), phosphate buffered saline (PBS), Tween 20, bovine serum albumin (BSA), and skim milk powder were from Merck KGaA (Darmstadt, Germany). 8-(4-Chlorophenylthio) adenosine-3′, 5′- cyclic monophosphate (8CPT-cAMP) was from BioLog (BioLog Life Science Institute GmbH & Co. KG, Bremen, Germany).

### Antibodies

Anti-PKA Catα (C-20) (sc-903; Santa Cruz Biotechnologies, Dallas, TX, USA), anti-PKA Reg I α/β (G-6) (sc-271446; Santa Cruz Biotechnologies), anti-PKA Reg II (L-16) (sc-26803; Santa Cruz Biotechnologies), anti-phospho-PKA substrate (RRXS*/T*)(100G7E) (#9624; Cell Signaling Technology, Danvers, MA, USA), anti-PP1 (E-9) (sc-7482; Santa Cruz Biotechnologies), anti PP2A α/β (ab32141; Abcam, Cambridge, UK), anti-LC3 I/II antibody (ABC929; Merck KGaA), anti-calreticulin (#2891S; Cell Signaling Technology), anti-GAPDH (sc-166574; Santa Cruz Biotechnologies), anti-total OXPHOS cocktail (ab110413; Abcam), anti-ATP synthase subunit α (ab14748; Abcam), and anti β-actin (A3854; Merck KGaA). Peroxidase-conjugated secondary antibodies were purchased from Merck or Santa Cruz Biotechnologies.

### Cell culture, cDNA constructs, and transfection

HT-29 and HeLa cells were grown at 37 °C in a 5% CO_2_ atmosphere in Dulbecco’s modified Eagle’s medium High Glucose (DMEM, Euroclone, Milan, Italy) supplemented with 10% fetal bovine serum (Euroclone), 100 U/ml penicillin, and 100 µg/ml streptomycin (Euroclone). Cells were tested for mycoplasma by polymerase chain reaction every 3–4 months. Cells were split every 2–3 days at a confluence of 80–90%. Twenty-four hours before transfection, HT-29 and HeLa cells were plated onto 15 mm diameter circular glass coverslips and were allowed to grow to 30% of confluence. Cells were transfected with Lipofectamine 2000 Reagent (Thermo Fisher Scientific, Waltham, MA, USA) following manufacturer’s instructions. Twenty-four hours after transfection, the coverslips were washed with supplemented DMEM. The experiments were performed 2 days after transfection. The ER-AKAR4 sensor was obtained by adding to the AKAR4 cDNA the cDNA coding for 27 N-terminal amino acids from cytochrome P450 (NCBI accession number AAA31436), excised by HindIII/BamHI digestion from ER-ABKAR, a gift from Takanari Inoue & Jin Zhang (Addgene plasmid #61508; http://n2t.net/addgene:61508; RRID:Addgene_61508. Addgene, Watertown, MA, USA) [29]. AKAR4 and AKAR4 Kras both cloned in pcDNA3 were a gift from Prof. Jin Zhang (Addgene plasmid #61621; http://n2t.net/addgene:61621; RRID:Addgene_61621); RIAD-mCherry, SuperAKAP-IS-mCherry, and scramble constructs were a gift of Prof. Alan Howe (University of Vermont). OMM-AKAR4, PKACα-mCherry, and PKIα-mCherry were previously generated in our laboratory [24, 27]. YFP-LC3 was a gift of Prof. Marco Sandri (University of Padova) and mCherry-GFP-LC3B was a gift from Robin Ketteler (Addgene plasmid #123230; http://n2t.net/addgene:123230; RRID:Addgene_123230).

### FRET imaging

HT-29 and HeLa cells plated onto glass coverslips and transfected with different FRET sensors were mounted onto an open perfusion chamber RC-25F (Warner Instruments, Hamden, CT, USA). The cells were perfused using a homemade gravity-fed perfusion system. The cells were bathed in Ringer’s modified buffer: NaCl 125 mM; KCl 5 mM; Na_3_PO_4_ 1 mM; MgSO_4_ 1 mM; Hepes 20 mM; glucose 5.5 mM; CaCl_2_ 1 mM; pH adjusted to 7.4 using 1 M NaOH. The experiments were performed on an Olympus IX81 inverted microscope (Olympus, Tokyo, Japan) equipped with a beam-splitter (Dual-ViewTM, Optical Insights, Santa Fe, New Mexico, NM, USA) and a CCD camera (F-ViewII, Soft Imaging System, Münster, Germany). The cyan fluorescent proteins (Cerulean for AKAR4 or mTurquoise for H187) were excited for 200 milliseconds at 430 nm, while the emission fluorescence was collected every 10–15 s for both donor and acceptor fluorophores at 480 and 545 nm, respectively. Automatic image collection and preliminary analysis were performed using the Cell R software (Olympus, Tokyo, Japan) and then analyzed with ImageJ plugin. Raw data were transferred to Excel (Microsoft, Redmond, WA, USA) for background subtraction and generation of the ratios, while graphs were generated by ImageJ or Prism software (GraphPad, San Diego, CA, USA).

### Confocal imaging

For the YFP-LC3 and mCherry-GFP-LC3 puncta images, populations of HT-29 or HeLa cells seeded onto 15 mm coverslips and expressing fluorescent light chain 3 (LC3) were rinsed twice with Ringer’s modified buffer and mounted onto an open perfusion chamber RC-25F (Warner Instruments, Hamden, CT, USA). Images were collected on a Leica TCS SP5 confocal scanning microscope using oil immersion 40x (HCX PL Apo lambda blue 40x/1.25 Oil UV, Leica, Wetzlar, Germany) or 60x (HCX PL Apo lambda blue 63x/1.40 Oil UV, Leica, Wetzlar, Germany) objectives.

### YFP-LC3 and mCherry-GFP-LC3 puncta analysis

The cells were seeded at low density onto 15 mm diameter glass coverslips and transfected with LC3-YFP, mounted onto an open perfusion chamber RC-25F (Warner Instruments), and bathed in Ringer’s modified buffer. For each coverslip, at least ten random images (approximately 15–20 cells) were acquired with Leica SP5 confocal microscope (Leica, Wetzlar, Germany) and LC3-YFP positive puncta were manually counted, while mCherry-GFP-LC3 analysis was performed using ImageJ as previously reported [30].

### Cell fractionation

Cells plated into 10 cm petri dishes and confluent 70–80% were washed with cold PBS and then suspended in cold Lysis Buffer (Qproteome Mitochondria Isolation Kit, Qiagen, Venlo, The Netherlands) complemented with protease (Bimake, Houston, TX, USA) and phosphatase inhibitors (Merck KGaA). Cells were incubated in continuous rotation for 10 min at 4 °C and then centrifuged at 1100 g for 10 min at 4 °C to precipitate permeabilized cells. The cell pellet was separated from the supernatant (which contained soluble proteins) and was resuspended in cold disruption Buffer (Qiagen, Venlo, The Netherlands) supplemented with phosphatase and protease inhibitors. The pellet was homogenized with 15–20 repeated aspirations with a blunt-end needle (gauge 26). The suspension was centrifuged at 1100 g for 5 min. The pellet containing the PNS fraction (nuclei, cell debris, and unbroken cells) was discarded. The supernatant was centrifuged at 7000 g for 10 min at 4 °C. The pellet containing the mitochondria-enriched fraction was resuspended in cold supplemented RIPA Buffer 1x (NaCl 150 mM; Tris-HCl 50 mM; NP-40 1%; sodium deoxycholate 0.5%; SDS 0.1%). The supernatant was enriched in the microsomal fraction.

#### Western blotting

The cells were lysed in cold RIPA buffer supplemented with protease inhibitors (Bimake) and phosphatases inhibitor cocktail (Merck KGaA). The cell lysates were cleared at 12.000 rpm at 4 °C for 12 min. The proteins (20–40 µg of total lysates or either 5–10 µg of the single fraction protein) were loaded onto 4–12% precast polyacrylamide gel (Bolt 4-12%, Bis-Tris plus gels; Thermo Fisher Scientific) for electrophoresis, and then transferred onto polyvinylidene fluoride (PVDF) membranes (Thermo Fisher Scientific). After, PVDF membranes were blocked for 1 h at room temperature in 5% (w/v) BSA-Tris-buffered saline/Tween 20 (TBST) for anti-phospho-antibodies or with 10% (w/v) non-fat-dry milk-TBST for non-phospho-antibodies. The membranes were then incubated overnight at 4 °C with continuous rotation with primary antibody (1:1000) in 1% BSA-TBST or 5% normal milk-TBST. The day after, membranes were washed four times with TBST at room temperature and then incubated for 1 h at room temperature with peroxidase-conjugated secondary antibodies. Peroxidase activity was detected with enhanced chemiluminescence (Luminata Crescendo Western HRP, Merck Millipore, Darmstadt, Germany). Anti-GAPDH or anti-β-actin antibodies were used as loading controls for the total cell lysates and to detect cytosol contamination in fractionation experiments, whereas OXPHOS antibody cocktail and calreticulin were used to determine mitochondrial and ER enrichment. Membranes were stripped using Restore Western Blot Stripping Buffer (Thermo Fisher Scientific) for 15 min at room temperature and then thoroughly washed with TBST. The densitometric analysis of immunoreactive bands was performed using ImageJ (https://imagej.nih.gov/ij/).

### Endogenous LC3B immunofluorescence

HT-29 cells were seeded on glass coverslips. After the indicated treatments (all treatments were performed for 24 h, while ChQ treatment was 4 h), cells were fixed with ice-cold methanol for 15 min, followed by three rinsing in PBS at room temperature. Fixed cells were then permeabilized with 0.3% Triton-X100 in PBS for 10 min and blocked in 5% normal goat serum, 0.2% Triton-X100 in PBS for 1 h. Staining with primary antibody LC3B 1:200 (Cell Signaling Technology #2775) was carried out overnight at 4 °C, followed by incubation with secondary antibody TRITC goat anti-rabbit 1:300 (T6778 Sigma) for 1 h at room temperature. Slides were mounted with Fluoroshield with DAPI (F6057 Sigma) and stored at 4 °C in the dark for subsequent acquisition. Acquisition was performed by confocal microscopy as indicated in the methods of the main manuscript.

### Statistical procedures

No statistical methods were used to determine sample size. Western blotting, confocal fluorescence imaging (YFP-LC3, mCherry-GFP-LC3, immunofluorescence) experiments were independently repeated at least three times with similar results (with the exception of endogenous LC3 immunofluorescence in which experiments were repeated twice as specified in the figure legend). FRET-based experiments were also repeated at least three times for a minimum number of ten cells. In case of lack of conclusiveness in statistical sub-significant trends, sample number was increased to more than three to improve statistical power. The exact number of cells for FRET experiments and the number of replicates are reported in the figure legends. The exclusion criteria for in vitro experiments were determined by the positive controls (FSK/IBMX or CPT) for phospho-PKA substrate western blots and FRET-based experiments in HeLa cells. For FRET-based experiments in HT-29 cells that do not respond to FSK/IBMX, we used two additional criteria, cell morphology and response to CalA treatment. The densitometric analysis of immunoreactive bands was performed using ImageJ (https://imagej.nih.gov/ij/). Data were analyzed with Microsoft Excel (Microsoft, Redmond, WA) or Prism (v8.3.0, GraphPad Software, San Diego, CA), while statistical analyses were carried out using Prism and KaleidaGraph software (Synergy Software, Reading, PA 19606). For comparisons involving only two groups of samples, statistical significance was evaluated by unpaired t-test. For comparisons involving more than two groups of samples, statistical significance was evaluated by ordinary one-way ANOVA followed by Bonferroni post-hoc test. In vitro experiments involving normalization of treated on untreated conditions, controls are expressed as percentages or fold ± SD (unless otherwise specified in the figure legend) calculated upon normalization on the average of raw control data of all experiments included in each analysis. Representative FRET traces are average of several cells ± SD from a single experiment (unless otherwise specified). A *p* value of <0.05 was considered statistically significant.

## Results

### Cyclic AMP signaling hinders the autophagic flux in HT-29 but not in HeLa cells

Our understanding of the role of cAMP signaling and its main effector PKA in autophagy remains incomplete; in fact, cAMP rises can be both inhibitory [12, 31, 32] and enhancing [12, 33], depending on the cell type. To clarify the mechanisms underpinning the effects of cAMP on autophagy, we performed a preliminary screening on different cell lines. Among those, we further tested HeLa (cervical cancer) and HT-29 (colon adenocarcinoma) based on their different autophagic responses to cAMP elevations. To investigate autophagy we used a fluorescent tool based on the microtubule-associated protein 1 LC3 [34]. LC3 is considered a reliable marker of autophagic progression and its fluorescent-tagged versions (YFP-LC3 [35, 36]) are used to measure autophagy.

**Fig. 1:**
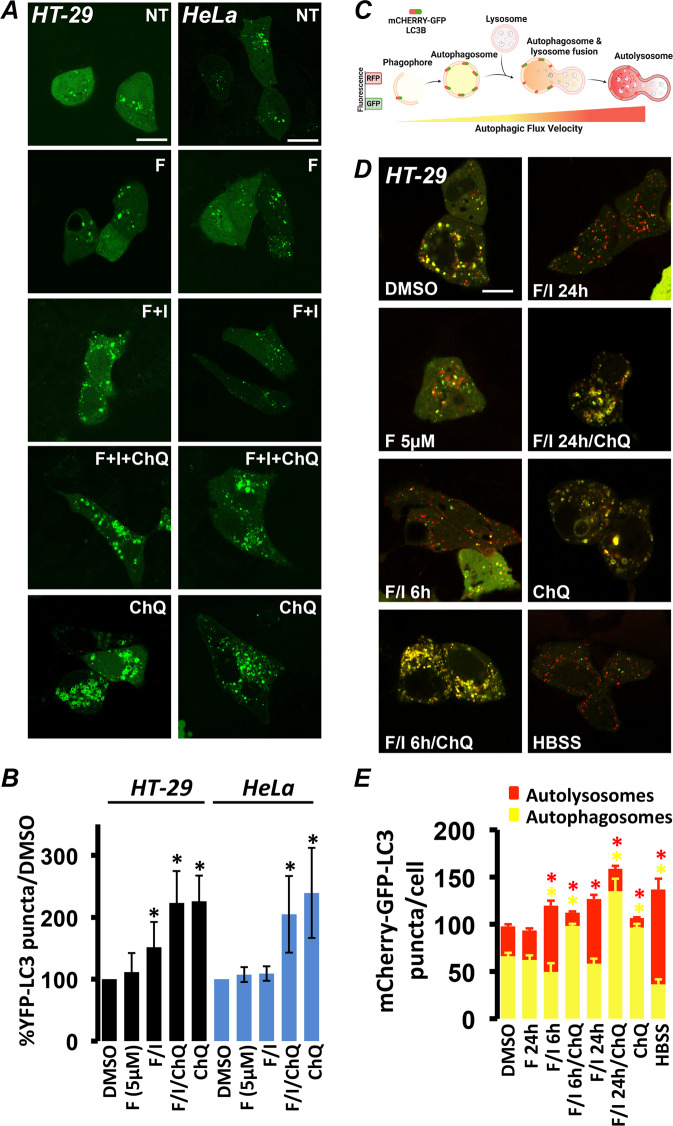
Cyclic AMP elevating agents increase the autophagic flux in HT-29 but not HeLa cells. **A** Confocal photomicrographs of HT-29 and HeLa cells expressing YFP-LC3. Treatment with 5 µM forskolin (F) did not alter the number of YFP-LC3-positive structures, while 20 µM F combined to 500 µM IBMX (F/I) increased YFP-LC3 puncta in HT-29 but not in HeLa cells. Chloroquine (ChQ) (100 µM) was used as control. **B** Summary of the effects on YFP-LC3 puncta of each treatment normalized to vehicle control (DMSO). Average of HT-29 cells: DMSO: 282, F 5 µM: 77, F/I: 276, F/I/ChQ: 31, ChQ: 38 in at least six independent experiments. Average of HeLa cells: DMSO: 98, F 5 µM: 58, F/I: 80, F/I/ChQ: 69, ChQ: 67 in at least four independent experiments. (*p < 0,04; **p < 0,01). **C** Schematic representation of how the sensor mCherry-GFP-LC3 works (created with BioRender.com). **D** Confocal photomicrographs of HT-29 cells expressing mCherry-GFP-LC3. Treatment with 5 µM forskolin (F) did not alter either the number or the balance between autophagosomes (mCherry^+^/GFP^+^) and autolysosomes (mCherry^+^/GFP^–^). Treatment with 20 µM F combined to 500 µM IBMX (F/I) for 6 or 24 h increased the number of autolysosomes and decreased the number of autophagosomes similarly to starvation (HBSS). Treatment with ChQ alone or in combination to F/I drastically decreased the number of autolysosomes and increased the number of autophagosomes. **E** Summary of the effects on mCherry-GFP-LC3 puncta number and type of each treatment. Average of HT-29 cells: DMSO: 83, F 5 µM: 38, F/I/6 h: 31, F/I/24 h: 37, F/I/6 h/ChQ: 12, F/I/24 h/ChQ: 15, ChQ: 40, HBSS: 22 in at least three independent experiments (*p < 0,01) (Scale bar 20 µm).

In cells expressing YFP-LC3, autophagosomes are visualized as fluorescent puncta or ring shaped structures [37]. We treated HeLa or HT-29 cells expressing YFP-LC3 with the adenylyl cyclase agonist FSK, or FSK together with the PDE-inhibitor IBMX for maximal stimulus. Twenty-four hours after treatment, cells were monitored using confocal microscopy and YFP-LC3 puncta were counted. As shown in Fig. 1A, B, treatment with FSK alone for 24 h had no significant effect on YFP-LC3 puncta compared to the vehicle control in both cell types, while treatment with FSK/IBMX increased the number of YFP-LC3 vesicles in HT-29 but not in HeLa cells. Both lines responded with an increase in the number of YFP-LC3 puncta when treated with ChQ, a drug that inhibits the fusion between autophagosomes and lysosomes [38] and has no effect on the cAMP/PKA axis (Fig. 1A, B). Exogenously expressed YFP-LC3 provides a valid estimate of autophagic flux variations; however, it also presents limitations [39, 40]. Therefore, we investigated the effects of cAMP on autophagy by comparing the levels endogenous LC3 using immunoblotting. LC3 free in the cytosol (LC3-I) is usually detected as a band at 16kD, while LC3-II is detected at 14kD due to the addition of phosphatidylethanolamine when bound to the autophagosome [41]. As shown in Supplementary Fig. S1A, treatment of HT-29 cells with FSK/IBMX or ChQ alone increased the intensity LC3-II and their combined treatment did not produce a cumulative effect. In addition, the increase in endogenous LC3 puncta in response to cAMP was confirmed by immunofluorescence experiments in HT-29 cells (Supplementary Fig. S1B). As expected, only ChQ increased LC3-II in HeLa cells (Supplementary Fig. S1C), confirming that in this model cAMP increases do not affect autophagy.

The results obtained with endogenous LC3 and YFP-LC3 suggest that, in HT-29 cells, cAMP affects autophagy; however, whether this treatment induced formation of new autophagosomes (increased autophagic flux) or blocked autophagosome degradation remained unclear. To better define the effects of cAMP, we employed an advanced LC3-based fluorescent probe, called mCherry-GFP-LC3 [30, 42, 43]. This construct takes advantage of the different pH sensitivities of GFP and mCherry (Fig. 1C). Being acid labile (pKa of 6.0 [44]), GFP is inert in acid environments and thus labels efficiently only neutral vesicles (autophagosomes), while mCherry is pH-stable (pKa 4.5 [45]) and can resist the autolysosomal environment (generated by the fusion of autophagosomes and lysosomes). Based on these characteristics, in cells that express mCherry-GFP-LC3, autophagosomes appear labeled by both GFP and mCherry, while autolysosomes are marked only by the latter [42, 43] (Fig. 1C). As shown in Fig. 1D,E, treatment of HT-29 cells expressing mCherry-GFP-LC3 with FSK did not affect either the total number of LC3-labeled vesicles or the ratio between autophagosomes and autolysosomes. On the contrary, treatment with FSK/IBMX for 6 or 24 h induced an increase in total vesicles. Qualitatively, both treatments resulted in a clear increase in autolysosomes accompanied by a decrease in autophagosomes. This trend was similar to that induced by starvation (HBSS) characterized by the decrease in GFP^+^ and the increase in mCherry^+^ vesicles as previously reported [46], and was reversed by treatment with ChQ, which resulted in a decrease in autolysosomes and increase in autophagosomes (Fig. 1D, E). To confirm the shift from autophagosomes to autolysosomes, we calculated the Pearson’s correlation coefficient between GFP and mCherry. We reasoned that the correlation of mCherry and GFP would be proportional to the number of autophagosomes (both proteins fluorescent) and inversely proportional to the number of autolysosomes (labeled only by mCherry). As shown in Supplementary Fig. S1D, Pearson’s coefficient was significantly lower after treatment with FSK/IBMX (both for 6 and 24 h) and after starvation (HBSS), while treatment with ChQ restored the correlation between mCherry and GFP (more double-labeled autophagosomes). Taken together, the above results clearly suggest that increases in cAMP in HT-29 cells result in the increase of the autophagic flux.

### PKA-dependent phosphorylation is hindered in HT-29 but not in HeLa cells

**Fig. 2:**
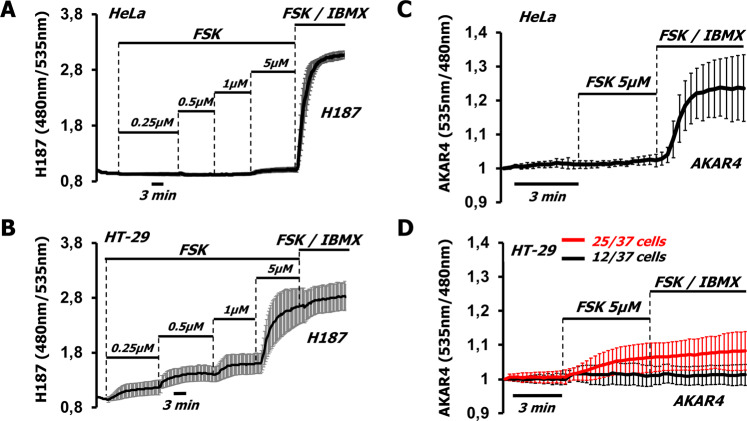
HT-29 cells display low PKA-dependent phosphorylation in response to high cAMP levels. **A** HeLa (averaged traces ± SD of six cells from one experiment) or **B** HT-29 cells (averaged traces ± SD of seven cells from one experiment) expressing the cAMP-sensitive FRET-based sensor H187 were challenged with increasing doses of forskolin (FSK) followed by FSK (20 µM) together with IBMX 500 µM (FSK/IBMX) to saturate the sensor. **C** HeLa cells expressing the PKA-dependent phosphorylation sensor AKAR4 marginally responded to FSK alone; however, addition of IBMX 500 µM produced a near-saturation response. Saturation was achieved by FSK/IBMX (averaged traces ± SD of 20 cells from four independent experiments). **D** In HT-29 cells AKAR4 gave extremely low responses independent of the treatment (averaged traces ± SD of 37 cells from 15 independent experiments).

The simplest explanation for the different effects of FSK/IBMX on autophagy is that these treatments do not produce the same cAMP levels in HeLa and HT-29 cells. To address this possibility, we used FRET-based sensors to measure cAMP in real time. In HeLa cells, the cAMP-sensitive sensor EPAC-S^H187^ (H187) [24, 25, 47] displayed small cAMP increases in response to FSK (Fig. 2A), which were greatly enhanced by addition of IBMX. When HT-29 cells were treated with the same protocol, H187 displayed grater responses, compared to HeLa, and was already nearly saturated by the addition of FKS alone, as demonstrated by the minimal effect of higher FSK concentrations (10 µM) together with IBMX (Fig. 2B). To compare the responses of H187 in the two models, we calculated the maximum range of the sensor, by subtracting the value of the ratio measured at time 0 (*R*_0_) from the maximum ratio (*R*_max_) registered by FSK/IBMX and found that H187 displayed the same dynamic range in both cell lines (Supplementary Fig. S2A). Taken together, these data indicate that in HT-29, FSK alone is sufficient to cause a major increase in cAMP, while in HeLa cells PDE inhibition is necessary to bring the cAMP levels to values indistinguishable from those attained in HT-29. Noteworthy, the ratio signal of H187 has been calculated to saturate at around 100 μM cAMP [24, 47], i.e., well above the levels of cAMP necessary to fully activate PKA [48].

The connection between cAMP and autophagy was attributed to PKA-dependent phosphorylation of components of the autophagic machinery [15]. To measure PKA-dependent phosphorylation, we transfected HeLa and HT-29 cells with the FRET-based sensor AKAR4 that provides an indirect estimate of PKA activity by monitoring the phosphorylation of a PKA consensus sequence [25, 28, 49]. As shown in Fig. 2C, in HeLa cells the kinetics of PKA activation mirrored the cAMP profile detected by H187. In fact, very little PKA-dependent phosphorylation was detected by FSK, and the AKAR4 signal quickly reached its peak value when we increased the level of FSK and added IBMX (see also [27]). Surprisingly, in HT-29 cells PKA-dependent phosphorylation did not correlate with the rises of cAMP. In fact, roughly 30% of the cells did not respond to any treatment, while the remaining 70% responded with modest AKAR4 signals when treated with FSK, and by further addition of IBMX (Fig. 2D). To test whether AKAR4 function was impeded in HT-29 cells we co-expressed catalytically active PKA tagged with the far-red fluorescent protein mCherry (PKA-Cα-mCherry) [24] together with the sensor. PKA-Cα elicited high AKAR4 signals in HT-29, similar to those observed in HeLa and other models [24, 25] using either FSK/IBMX or PKA overexpression, demonstrating that the sensor is fully functional also in HT-29 (Supplementary Fig. S2B), and suggesting that PKA activity in this model cannot fully phosphorylate the sensor. In addition, expression of the specific PKA inhibitor protein kinase inhibitor [24] resulted only in a slight decrease of the starting ratio, indicating that AKAR4 was not saturated by basal PKA activity (Supplementary Fig. S2B).

We next used the cell permeable cAMP analog 8CPT-cAMP that can efficiently activate PKA [27]. 8CPT-cAMP is conjugated to an acetoxymethyl ester group to facilitate permeability, and, as shown in Supplementary Fig. S3A, doses as low as 5 µM saturate H187 in HT-29 cells. To make sure we would achieve maximum intracellular concentration we used 10 µM; however, no appreciable increase of AKAR4 ratio in HT-29 cells was registered (Supplementary Fig. S3B). To exclude the possibility that our observations were valid only for HT-29 cells, we purchased and tested a cell line called HT-29-MTX [50]. HT-29-MTX cells are considered a gastric model, since they were obtained after methotrexate treatment of HT-29 cells and selection on the basis of gastric cell characteristics [51]. As shown in Supplementary Fig. S3C, HT-29-MTX cells produced high levels of cAMP in response to FSK/IBMX treatment; however, the responses of AKAR4 (Supplementary Fig. S3D) were similar to those of HT-29. Taken together, the data of Figs. 1 and 2 demonstrate that in HeLa cells autophagy is unaffected by large increases in cAMP that induce a massive activation of PKA-dependent phosphorylation of AKAR4; on the contrary, in HT-29 cells, similar, very large increases in cAMP cause a significant effect on autophagy, despite a modest increase in PKA-dependent phosphorylation of the same probe.

### HT-29 cells express PKA and display limited PKA substrate phosphorylation in response to cAMP

**Fig. 3:**
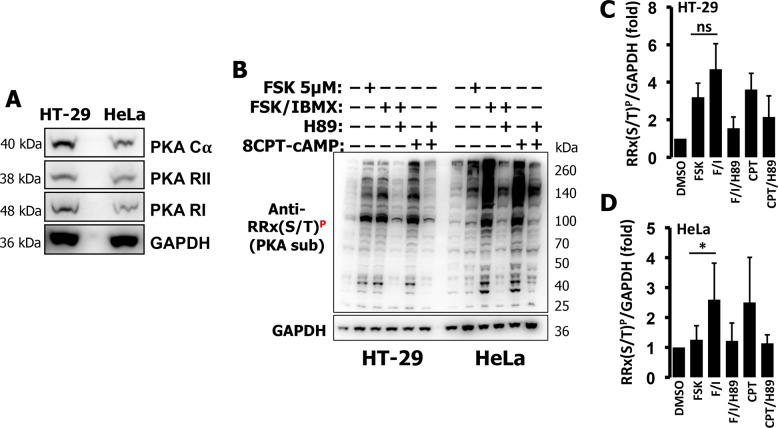
HT-29 and HeLa cells have comparable PKA levels but differ in PKA-dependent phosphorylation. **A** Western blotting of PKA components in total cell lysates of HT-29 and HeLa cells. Both cell lines expressed similar levels of PKA catalytic subunit (PKA Cα) as well as PKA regulatory subunits I and II (PKA RII and PKA RI). GAPDH was used as loading control. Representative data of three independent experiments. **B** Western blotting of phospho-bands assessed by a phospho-PKA substrate antibody, RRX(S/T)^P^ of total cell lysates (40 µg) of HT-29 and HeLa cells treated with FSK alone (5 µM), FSK (20 µM) combined with IBMX (500 µM), 8CPT-cAMP (5 µM), or the PKA inhibitor H89 (30 µM). **C** Intensities of phospho-bands, normalized to GAPDH, for HT-29 and HeLa (**D**). Averages ± SD from at least three independent experiments.

We then set to investigate the cause of the low PKA-dependent phosphorylation observed in HT-29 cells. We tested PKA expression and found comparable levels of PKA Cα, PKA RI, and PKA RII in both cell lines (Fig. 3A). To detect PKA-dependent phosphorylation of endogenous substrates, we used an antibody able to recognize phosphorylated PKA substrates (RRX(S/T)^P^). As shown in Fig. 3B (left side) and quantified in Fig. 3C, HT-29 cells treated with FSK 5 µM displayed an increase in PKA-dependent phosphorylation that was not significantly augmented by increasing the levels of FSK (to 10 µM), the addition of IBMX or treatment with high doses of 8CPT-cAMP. As expected, the increases in phosphorylation of endogenous substrates were almost completely abolished by the PKA inhibitor H89. HeLa cells responded with low PKA activity to FSK and this response was dramatically increased by addition of IBMX. Interestingly, when the HeLa cells were treated with 8CPT-cAMP (which is PDE-resistant) the response was similar to that of FSK/IBMX (Fig. 3B right side and Fig. 3D). These findings are in line with the data obtained using AKAR4 (Fig. 2C) and confirm that PDEs are major regulators of the cAMP/PKA axis in HeLa cells.

### PKA-dependent phosphorylation is highly compartmentalized in HT-29 cells

**Fig. 4:**
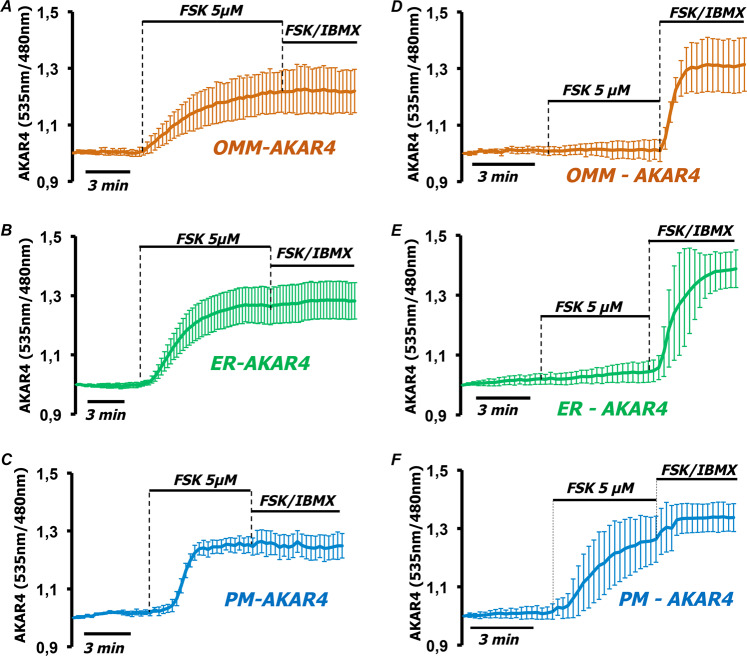
PKA-dependent phosphorylation remains compartmentalized in HT-29 but not in HeLa cells at high cAMP levels. HT-29 cells expressing AKAR4 constructs targeted to **A** the outer mitochondrial membrane (OMM-AKAR4), **B** the endoplasmic reticulum facing the cytosol (ER-AKAR4), and **C** the plasma membrane (PM-AKAR4). All constructs responded to treatment with FSK 5 µM, while addition of FSK 20 µM combined to IBMX 500 µM did not further affect the FRET ratio, indicating that all sensors were saturated. Averaged traces ± SD of 15 cells in four independent experiments for OMM-AKAR4; 13 cells in four independent experiments for ER-AKAR4 and 4 cells in three independent experiments for PM-AKAR4. **D** HeLa cells expressing OMM-AKAR4, **E** ER-AKAR4, and **F** PM-AKAR4. Only PM-AKAR4 responded to treatment with FSK 5 µM, while all constructs saturated with FSK 20 µM combined to IBMX 500 µM. Averaged traces ± SD of 13 cells in three independent experiments of OMM-AKAR4; 10 cells in three independent experiments of ER-AKAR4, and 10 cells in three independent experiments for PM-AKAR4.

The AKAR4 sensor mirrors PKA-dependent phosphorylation of soluble targets but underestimates that of substrates bound to subcellular structures. To address this issue we used three targeted versions of AKAR4, to the outer mitochondrial membrane (OMM-AKAR4) [24, 25], the endoplasmic reticulum facing the cytosol (ER-AKAR4), and plasma membrane (PM-AKAR4) [28]. The correct localization of the sensors was confirmed by fluorescence microscopy (Supplementary Fig. S4A). As shown in Fig. 4, in HT-29 cells all targeted sensors responded with a strong rise to FSK alone while further addition of IBMX had marginal additional effects on the responses of OMM-AKAR4 (Fig. 4A), ER-AKAR4 (Fig. 4B), or PM-AKAR4 (Fig. 4C). In HeLa cells, targeted sensors reached saturation by high levels of cAMP [27, 28]. Indeed, FSK together with IBMX induced in HeLa cells maximal ratio changes both in OMM-AKAR4 and ER-AKAR4 (Fig. 4D–F). Interestingly, 5 µM FSK induced the activation of PKA only near the plasma membrane in HeLa cells. This was not surprising since FSK activates plasma membrane adenylyl cyclases resulting in high local cAMP increases difficult to control by PDEs. To exclude the possibility that AKAR4 responses differ due to the targeting we calculated the maximum ratio (*R*_max_) reached by each targeted sensor in HT-29 cells and compared it to the *R*_max_ achieved by cytosolic AKAR4 in HT-29 and HeLa cells. As shown in Supplementary Fig. S4B, the *R*_max_ of localized sensors is similar to the *R*_max_ of soluble AKAR4 in HeLa cells and much higher than it is in HT-29.

### In HT-29 cells phosphatases topologically shape PKA-dependent phosphorylation independently of the cAMP levels

**Fig. 5:**
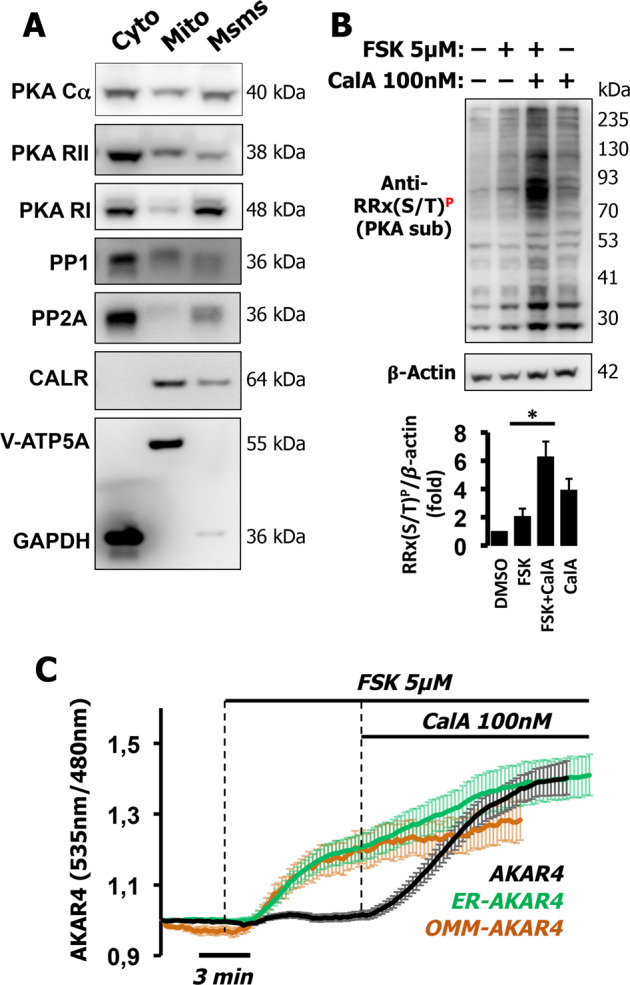
Soluble phosphatases shape PKA-dependent phosphorylation in HT-29 cells. **A** Western blotting of PKA components and phosphatases in cytosolic/soluble (cyto), mitochondria-enriched (Mito), and microsomal-enriched (Msms) fractions from HT-29 cells. An antibody mixture against ATP synthase subunit alpha (V-ATP5A) and GAPDH assessed mitochondrial enrichment and cytosol, respectively, while calreticulin (CALR) was used to determine ER enrichment. **B** Total cell lysates (40 µg) of HT-29 were treated with FSK (5 µM), Calyculin A 100 nM (CalA), or both combined and the phosphorylation status of endogenous PKA substrates was assessed by the phospho-PKA substrate-specific antibody, RRX(S/T)^P^. Combination of CalA and FSK produced a dramatically greater responses than those of the individual drugs alone, indicating an additive effect as summarized in the quantification histogram (inset). β-actin was used as loading control. Experiments were repeated at least three times. **C** HT-29 cells expressing AKAR4 (black), OMM-AKAR4 (orange), or ER-AKAR4 (green) challenged with FSK (5 µM) followed by CalA (100 nM) to block phosphatases. Averaged traces ± SD of 12 cells for AKAR4, 15 cells for OMM-AKAR4, and 12 cells for ER-AKAR4, from three independent experiments for each sensor.

We previously demonstrated that local PKA-dependent phosphorylation events largely depend on the activity and localization of phosphatases [24, 25]. To test whether this mechanism could explain our observations, we fractionated HT-29 cells and found that phosphatases were mainly present in the soluble/cytosolic fraction, while PKA subunits were distributed equally between cytosol, mitochondria, and microsomes (Fig. 5A) as previously observed [24, 27]. We next tested the effect of phosphatase inhibition on the phosphorylation of PKA targets by western blotting. As expected, treatment of HT-29 cells with FSK alone produced a slight increase in the overall phosphorylation; however, phosphatase inhibition with CalA dramatically increased the responses of FSK to levels much higher than those achieved by FSK or CalA alone (Fig. 5B and inset). We next tested the effects of CalA on PKA-dependent phosphorylation on AKAR4-derived sensors. As summarized in Fig. 5C, both OMM-AKAR4 and ER-AKAR4 responded to FSK, while the soluble AKAR4 responded only when CalA was added in addition to FSK.

### In HT-29 cells the effects of cAMP on autophagy depend on localized PKA type II moieties

**Fig. 6:**
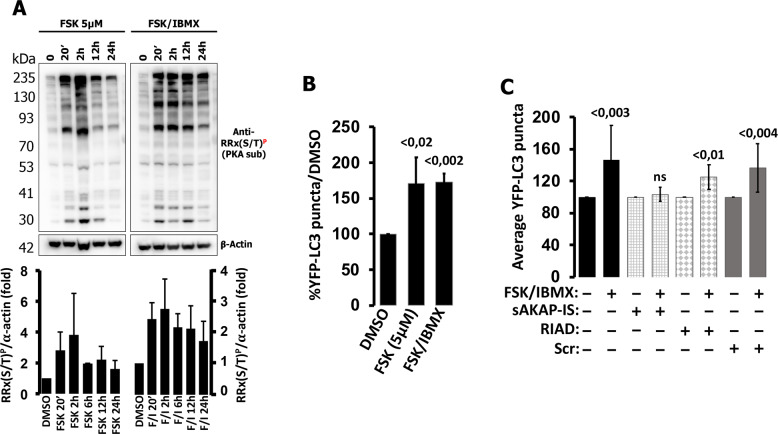
Localized PKA RII is responsible for the effects of cAMP on autophagy. **A** HT-29 cells were challenged with FSK (5 µM) or FSK (20 µM) combined to IBMX (500 µM) and total cell lysates were prepared at different timepoints. The phosphorylation status of endogenous PKA substrates was assessed by a phospho-PKA substrate-specific antibody, RRX(S/T)^P^ and is summarized in the inset (bar graphs: average ± SD of three independent experiments). **B** Summary of the effects on YFP-LC3 puncta of FSK 5 µM or FSK 20 µM combined to IBMX (500 µM) treatments, normalized to vehicle control (DMSO), measured after 4–6 h after treatment. Average of at least three independent experiments. **C** Summary of the effects of super AKAP-IS-mCherry (sAKAP-IS), RIAD-mCherry (RIAD), and scramble-mCherry (scr) on the number of YFP-LC3 puncta generated by FSK (20 µM)/IBMX (500 µM). Data presented are average of four independent experiments for sAKAP-IS, RIAD, and scramble, and ten experiments for control (15–25 cells for each treatment).

During our FRET experiments we noticed that in HT-29 cells both cAMP levels (measured by H187) and PKA-dependent phosphorylation of membrane-bound substrates reached saturating values with FSK, and that inhibition of PDEs by IBMX produced only marginal additional effects (Figs. 2B, 3B, and 4A–C). Based on these findings, we expected that FSK and FSK/IBMX would have similar effects on autophagy; however, contrary to FSK/IBMX, FSK alone did not affect autophagy after 24 h (Fig. 1). We reasoned that this discrepancy could be explained by the length of the treatment. In fact, FRET experiments were performed in real time and cells were monitored for several minutes, while the effects on autophagy were measured 18–24 h after treatment. We thus set to test whether the patterns of PKA-dependent phosphorylation produced by FSK or FSK combined with IBMX changed in relation to time. We challenged HT-29 cells and tested for total PKA substrate phosphorylation at different timepoints spanning from 20 min to 24 h. As shown in Fig. 6A and inset, PKA-dependent phosphorylation induced by FSK reached its maximum peak after 2 h and gradually subsided 12–24 h after treatment, while phosphorylation induced by FSK/IBMX reached its maximal peak between 20 min and 2 h and the phosphorylation patterns persisted for 24 h. Similar experiments in HeLa cells showed that also in this model addition of IBMX resulted in persistent long-term PKA-dependent phosphorylation of general targets (Supplementary Fig. S5A–C). These experiments demonstrated that PKA activity upon FSK treatment subsides due to the actions of PDEs since the addition of IBMX expanded the duration of PKA target phosphorylation. Based on these results we hypothesized that the effects of FSK alone on autophagy in HT-29 cells would be visible mainly at shorter timepoints; accordingly, we treated the cells expressing YFP-LC3 for 4–6 h and found that FSK had the same effects as FSK/IBMX on LC3 puncta formation (Fig. 6B).

PKA compartmentalization is mainly achieved by the binding of the holoenzyme to AKAPs through its regulatory subunits. Interestingly, AKAPs can selectively tether RI or RII, and in some instances both (dual specificity AKAPs), accounting for the subcellular distribution of the different PKA types [52]. Since in HT-29 cells PKA-dependent phosphorylation is confined in close proximity of membranes (Fig. 4) we set to test whether AKAP-PKA signalosomes were involved in the regulation of autophagy. To investigate the contribution of type I or type II PKA we took advantage of two peptides designed to interfere selectively with the interaction between AKAPs and PKA-RII (super AKAP-IS [53, 54]) or AKAPs and PKA-RI (RIAD [55]). We co-expressed super AKAP-IS, RIAD, or a scramble peptide all tagged with mCherry [56] together with YFP-LC3. One day after transfection we treated the cells with FSK/IBMX or vehicle (DMSO) and 18–24 h later we counted the number of YFP-LC3 puncta. As summarized in Fig. 6C, co-expression of RIAD and the scramble peptide did not modify the effects of FSK/IBMX on YFP-LC3 puncta formation; however, when super AKAP-IS was expressed, the effect was fully abolished. These experiments provided a link between the compartmentalization of RII-based PKA holoenzymes and the regulation of autophagy in HT-29 cells.

## Discussion

Under normal conditions autophagy targets obsolete or damaged organelles and protein aggregates [2], and it is considered to be a finely regulated process with a high degree of spatiotemporal compartmentalization [57, 58]. Autophagy is regulated by proteins that can undergo post-translational modifications [9, 11, 59] enabling the autophagic process to be initiated at any subcellular location within a small timeframe. For autophagy to occur in a specific subcellular locus, the core components of its regulatory network must be coordinated; however, the molecular pathways that deliver regulatory inputs are not fully explored. Here we show that localized PKA signaling is involved in the regulation of the autophagic flux. cAMP has been shown to regulate autophagy both in yeast and mammals [60–62]. Mainly, its effects depend on PKA and its ability to interfere with autophagy, indirectly [61, 63] or directly, by phosphorylating core components of the autophagic machinery. PKA can phosphorylate several autophagy-related proteins (Atgs) [15, 64], inhibit the key autophagic regulator mTORC1 by phosphorylating RAPTOR and even phosphorylate LC3 [31]. In addition, recent data suggested a dynamic crosstalk between autophagy and PKA in yeast [65, 66]. While a cardinal characteristic of PKA is compartmentalization, the effects of localized PKA events on autophagy have not been investigated. Here we found that increases in cAMP affect autophagy in HT-29 but not in HeLa cells. By investigating the molecular aspects of the cAMP/PKA axis we discovered that, contrary to HeLa, PKA in HT-29 cells is inefficient at phosphorylating soluble exogenous (AKAR4) or endogenous substrates, while it is highly effective in phosphorylating substrates at the proximity of membranes (mitochondria, endoplasmic reticulum, plasma membrane) independently of cAMP gradients. We have recently demonstrated that PKA-dependent phosphorylation can be spatiotemporally confined in a cAMP-independent manner, by phosphatases [24, 25]. In line with this, we found that phosphatases are responsible for PKA confinement also in HT-29 cells. However, as compared to HeLa cells [27] and a cardiac model [24, 25], where massive and homogeneous increases in cAMP levels overcome the restrictive effects of phosphatases [25], in HT-29 cells the confinement of PKA-dependent phosphorylation appears to be completely dependent on phosphatases (Figs. 1 and 4). Taking advantage of this highly compartmentalized model, we used specific peptides to selectively perturb the tethering of PKA type I and type II. We found that disengagement of PKA type II from its AKAPs completely abolished the effect of cAMP in autophagy, while displacement of PKA type I had no effect. These data offer a molecular link between localized RII-PKA signaling and autophagy. It is noteworthy that RIα subunits were shown to complex with late endosomes and autophagomes; however, their effects did not seem to rely on target phosphorylation [67].

The autophagic process begins with the formation of an isolation membrane (phagophore) that engulfs the cargo and eventually matures in the autophagosome. LC3 is important for both the elongation of the isolation membranes and the maturation of the autophagosome and is present in both these structures [68]. Comparing the effects of cAMP to those of starvation (induced autophagy) or ChQ (inhibited autophagy), we established that PKA has an effect on autophagy that resembles that of starvation. Discriminating between autophagosomes and autolysosomes we established that local PKA activation resulted in increased autolysosomes and decreased autophagosomes, it is tempting therefore to speculate that local PKA moieties could facilitate the fusion between lysosomes and autophagosomes to generate autolysosomes. However, it is not possible to exclude that this pathway may be involved in the processes that regulate membrane availability of the nascent autophagosomes. In fact, multiple organelles could act as membrane sources for autophagosomes, including the mitochondria [69], the plasma membrane [70], and the endoplasmic reticulum [71], all sites we found high PKA responses in HT-29 cells. Based on our finding that plasma membrane PKA is activated also in HeLa cells by FSK (Fig. 4F), it is more likely that PKA bound at the outer mitochondrial membrane or the ER would be responsible for the effects we observed. The future challenge will be to establish the precise PKA moieties that are able to regulate autophagy and most importantly, the targets participating in this process, all information that will enable us to exploit this novel regulatory mechanism to control autophagy by “surgically” targeting specific AKAP-PKA signalosomes.

## Supplementary information

Supplemental Information

## Data Availability

All materials will be provided under Materials Transfer Agreement upon request to KL (Konstantinos.lefkimmiatis@unipv.it).
