# Supplementary material for: PKA compartmentalization links cAMP signaling and autophagy
Source: Cell Death Differ. 2021 Mar 19;28(8):2436–49. doi: 10.1038/s41418-021-00761-8 (PMC8328970; doi:10.1038/s41418-021-00761-8)
Supplement: Supplementary file 1 — Supplemental Information [file 41418_2021_761_MOESM1_ESM.docx]

**Supplementary Information_ Grisan et. al.**

**Supplementary figures and figure Legends**

**
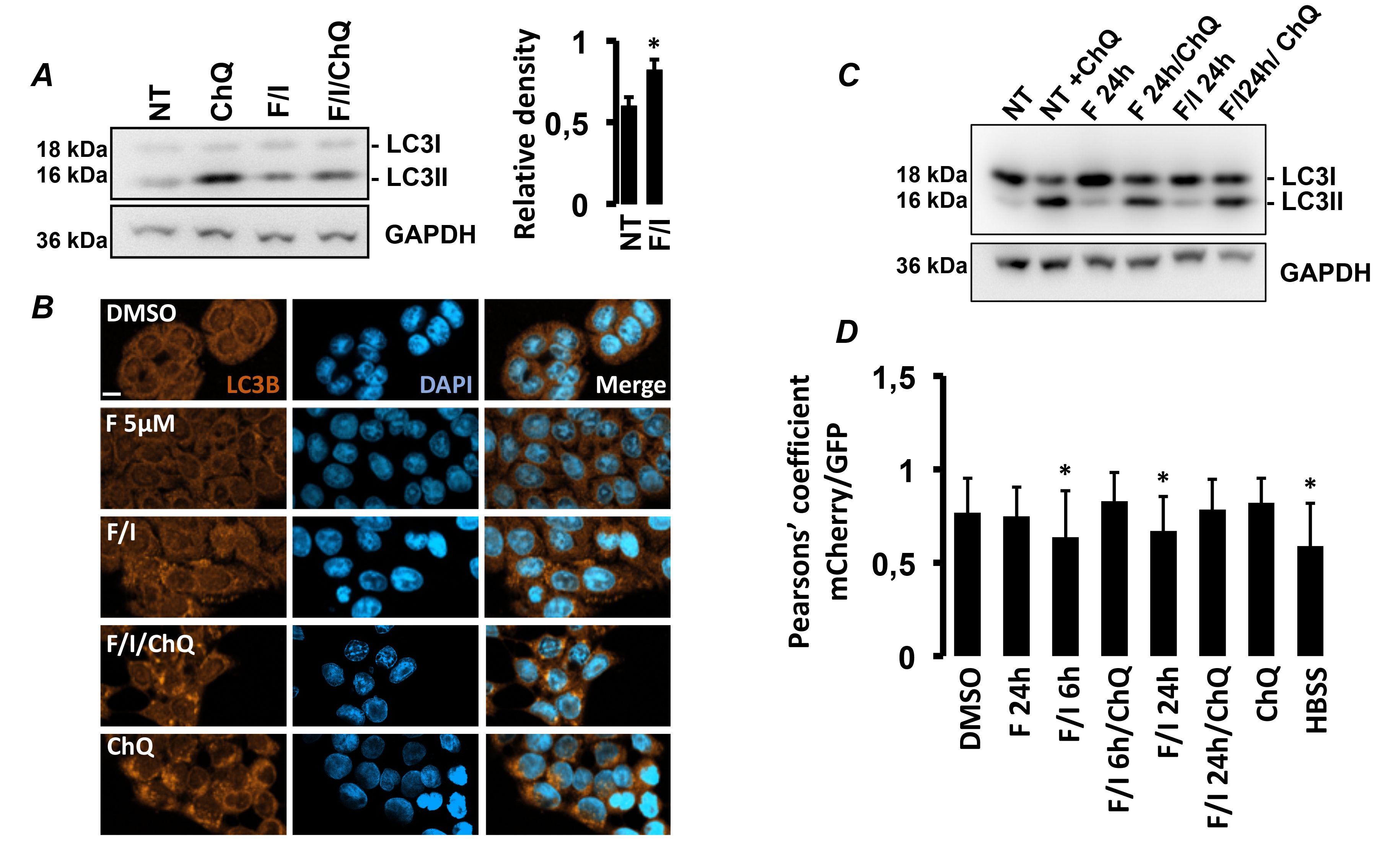
**

**Suppl. Figure 1**

*Increases in cAMP affect autophagy in HT-29 but not HeLa cells.*

**A)** Western blotting and quantification of endogenous LC3 in total cell lysates of HT-29 cells challenged with forskolin 20 µM combined to IBMX 500µM (F/I) alone or combined to Chq. GAPDH was used as control. Experiments were repeated at least 3 times. **B)** Immunofluorescence of endogenous LC3 in HT-29 cells. Treatment with FSK 5µM for 24h did not increase LC3 puncta. Treatment with F/I increased LC3 puncta, while addition of ChQ to F/I or alone increased both the volume and the number of LC3 positive puncta (representative of 2 independent experiments) (Scale bar 10µm). **C)** Western blotting of endogenous LC3 in total cell lysates of HeLa cells challenged with FSK alone or F/I with or without chloroquine. GAPDH was used as loading control. Experiments were repeated at least 4 times. **D)** Pearsons’ coefficient of mCherry-GFP-LC3 construct in HT-29 cells treated with F (24h), F/I (6h & 24h), F/I/ (6h & 24h)/ ChQ, HBSS or ChQ alone for 5hours. A decrease in the pearsons’ coefficient indicated a change in the balance between autophagosomes and autolysosomes. As expected only treatments with F/I (6 and 24h) and HBSS significantly decreased pearsons’ coefficient, indicating an increase in autolysosomes (mCherry^+^/GFP^-^). Pearsons’ coefficient was calculated in the same HT-29 cells of **fig. 1D** and **E**: DMSO: 83, F: 38, F/I/6h: 31, F/I/24h: 37, F/I/6h/ChQ: 12, F/I/24h/ChQ: 15, ChQ: 40, HBSS: 22 in at least 3 independent experiments. (* p<0,02).


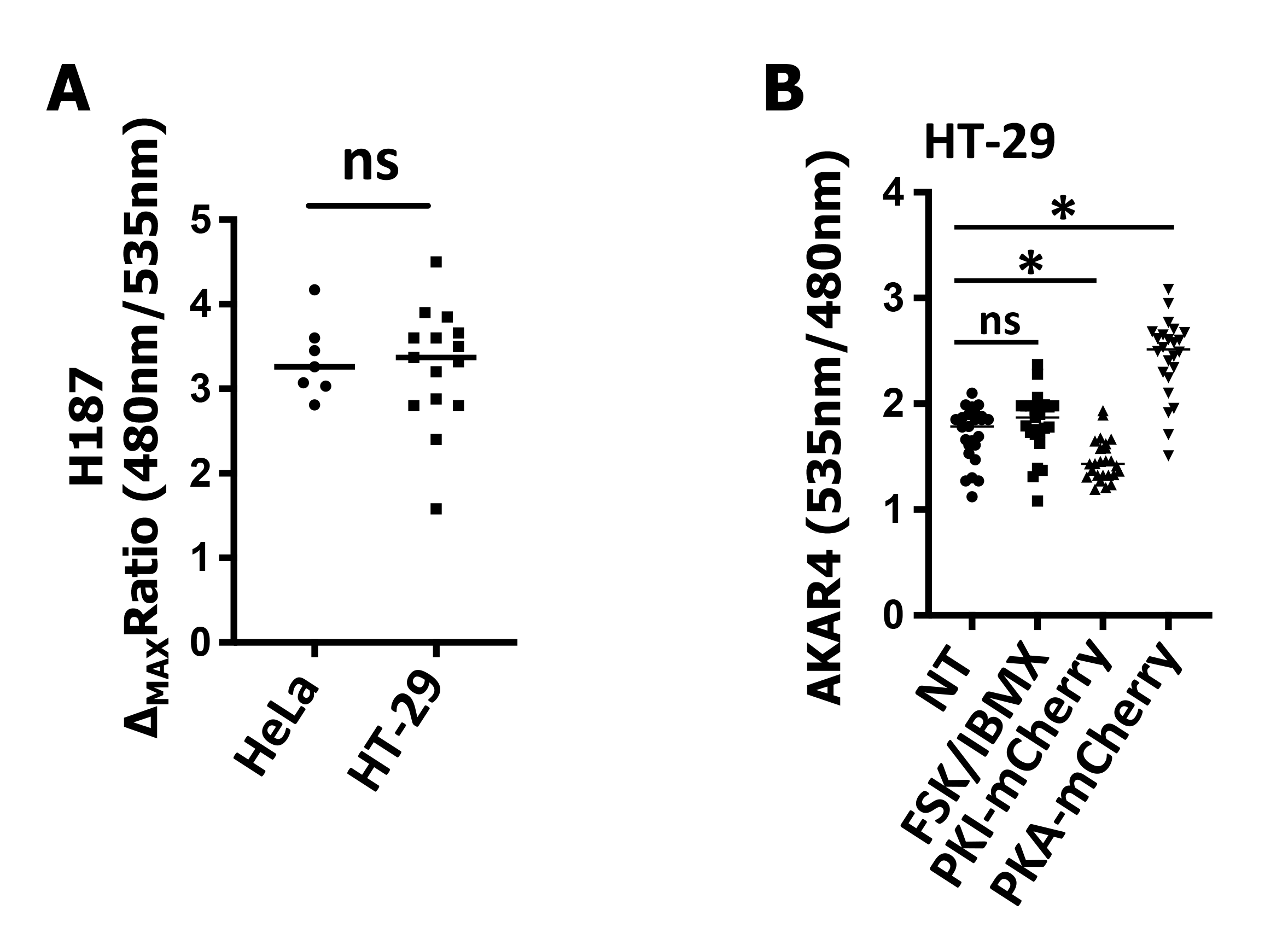


**Suppl. Figure 2**

*The FRET-based sensor for cAMP (H187) has similar dynamic ranges both in HT-29 and HeLa cells; the PKA-dependent phosphorylation (AKAR4) sensor is fully functional in HT29 cells*

**A)** Comparison of the maximal dynamic range reached by the cAMP FRET sensor H187 in HeLa and HT-29 cells. **B)** Maximum ratio reached by AKAR4 in HT-29 cells in response to FSK 20µM combined to IBMX 500µM, or by overexpressing a constitutively active PKA catalytic subunit (PKA-mCherry) or a PKA inhibitor (PKI-mCherry). Average of 4 experiments (* p <0,001).


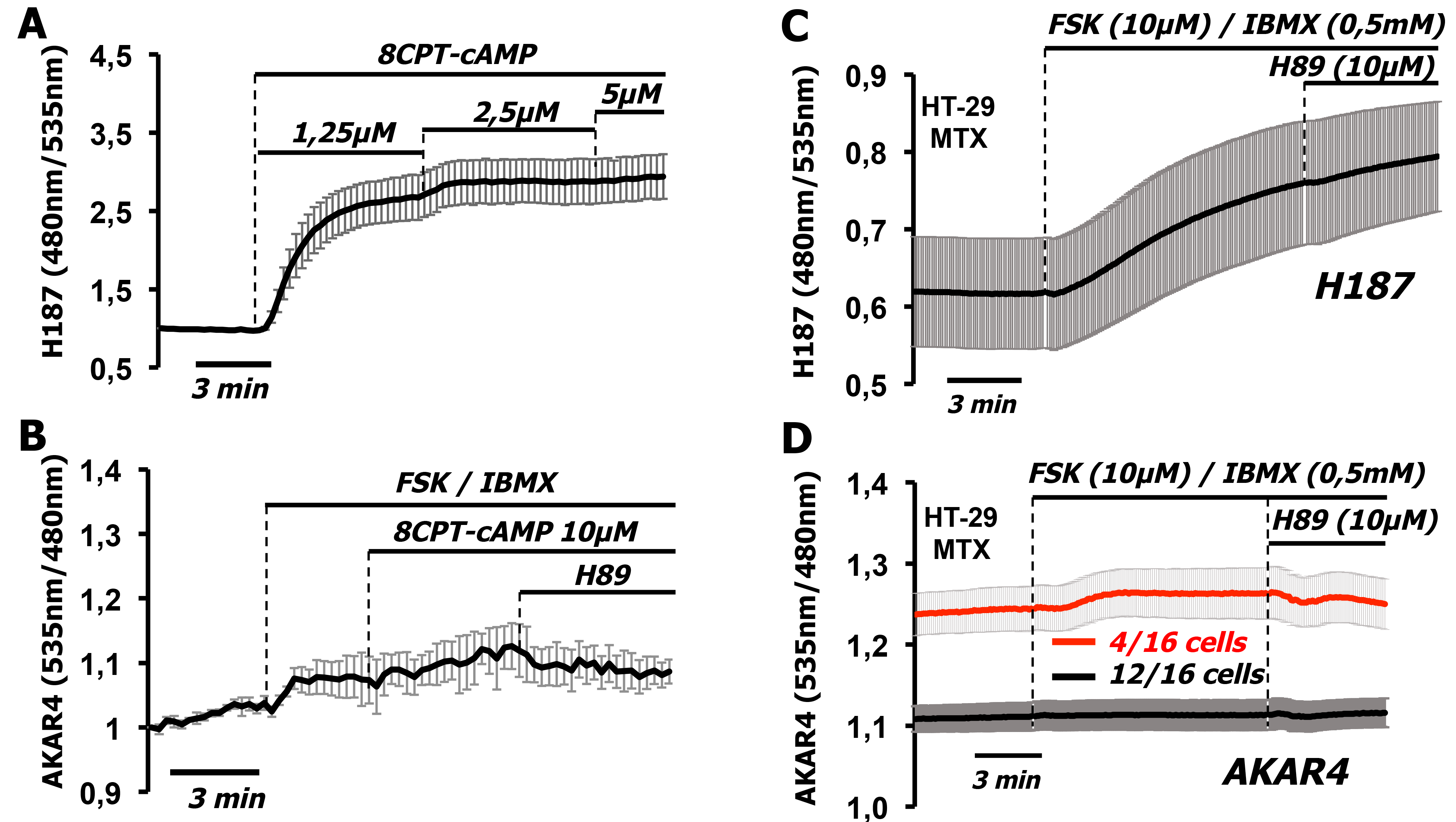


**Suppl. Figure 3**

*PKA-dependent phosphorylation is limited in HT-29 and HT-29-derived cell lines*

**A)** HT-29 cells expressing the cAMP-sensitive FRET-based sensor H187 were challenged with increasing doses of 8-CPT-cAMP. **B)** HT-29 cells expressing the PKA-dependent phosphorylation sensor AKAR4 marginally responded to 8CPT-cAMP (10 µM) or FSK (20 µM) combined to IBMX (500 µM). HT-29-MTX cells expressing H187 **(C)** or AKAR4 **(D)** challenged with FSK/IBMX. Average traces ± SEM of 4-6 cells from one representative experiments, each protocol was repeated at least 3 times.


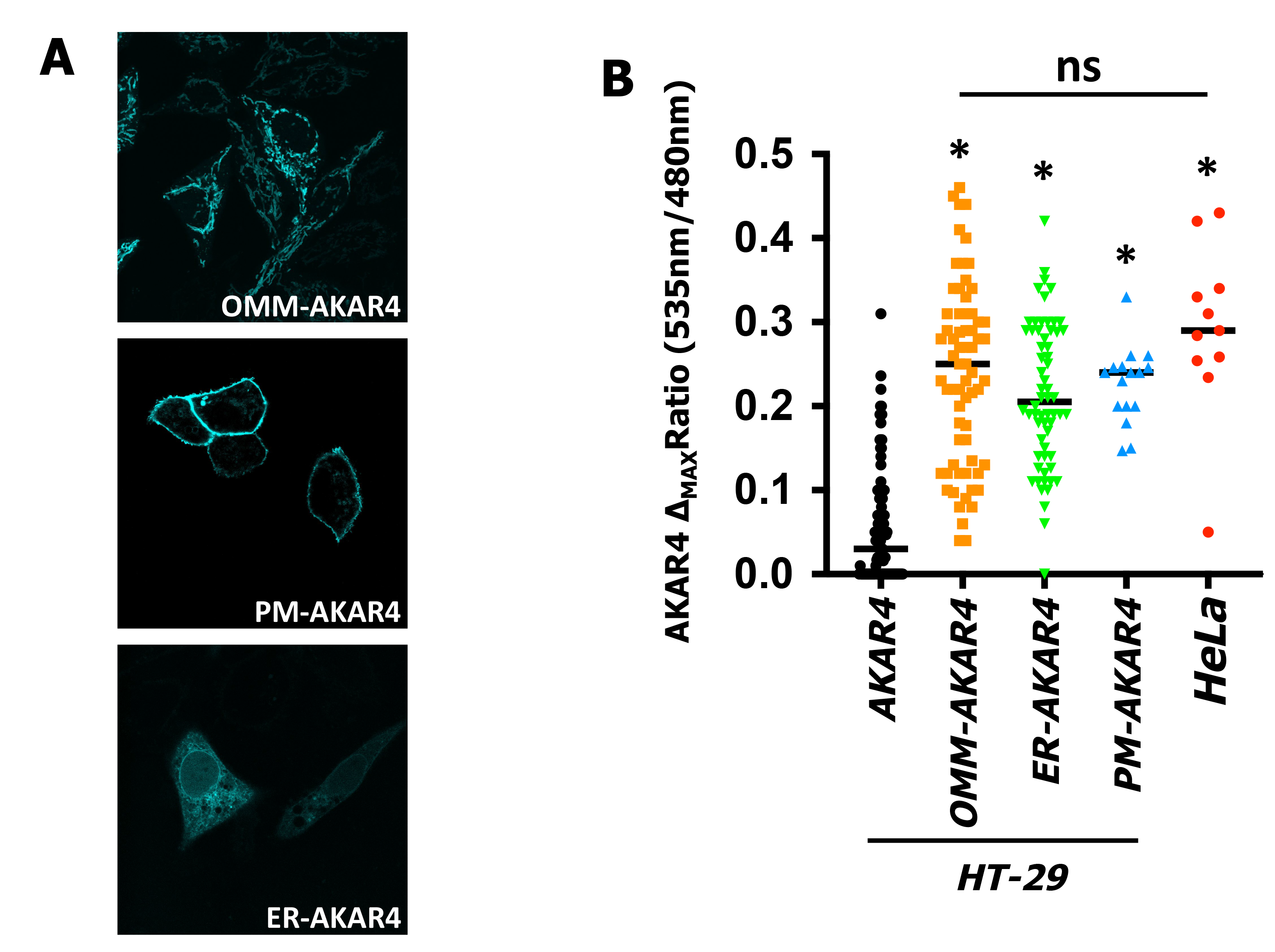


**Suppl. Figure 4**

*Targeted AKAR4 versions in HT-29 cells reach maximum ratios comparable to soluble AKAR4 in HeLa cells.*

**A)** Pseudo color images of OMM-AKAR4, PM-AKAR4 and ER-AKAR4. **B)** Comparison of the maximal dynamic range reached by soluble and targeted versions of the PKA-dependent phosphorylation FRET sensor AKAR4 in HT-29 with soluble AKAR4 in HeLa cells. Average of 6 experiments (* p <0,001).


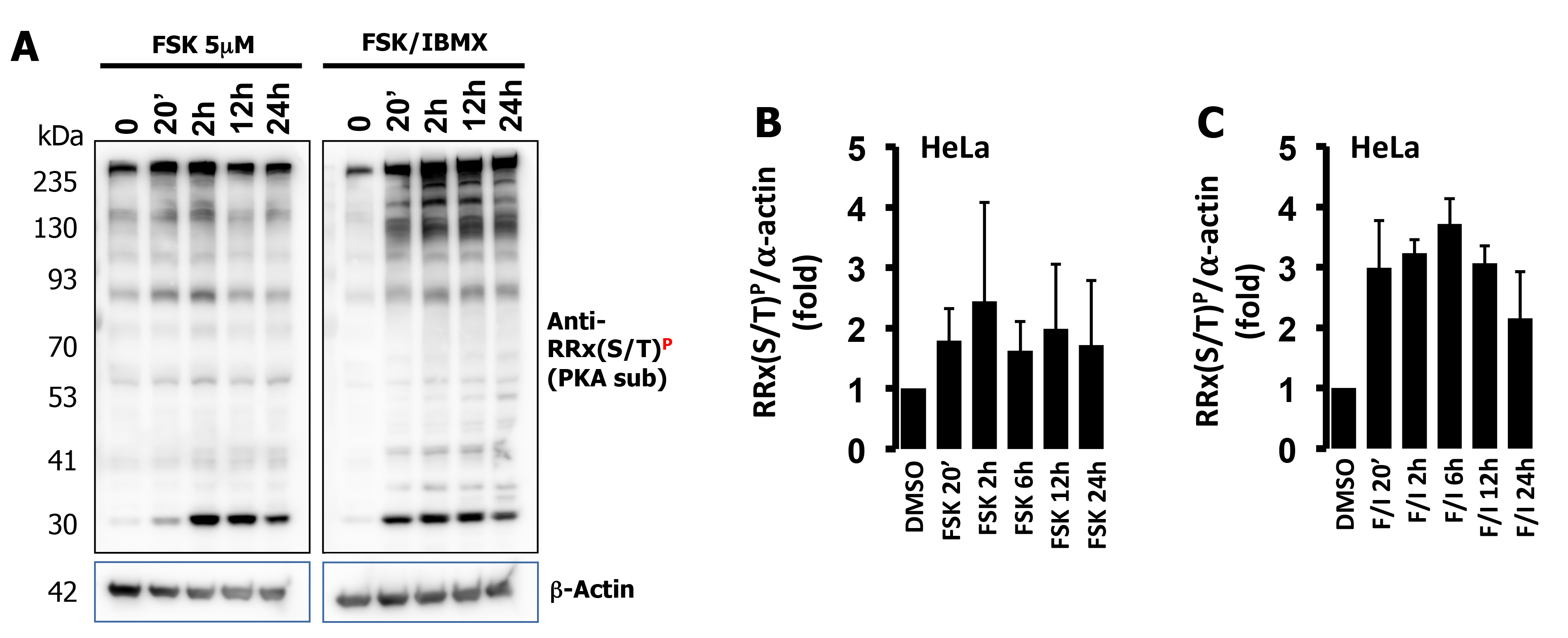


**Suppl. Figure 5**

*Time course of PKA-dependent phosphorylation levels during long incubations in HeLa cells.*

**A)** HeLa cells were challenged with FSK 5 µM or FSK 20 µM combined to IBMX 500µM (F/I) and total cell lysates were prepared at different timepoints. The phosphorylation status for FSK and F/I of endogenous PKA substrates was assessed by a phospho-PKA substrate specific antibody, RRX(S/T)^P^ and is summarized in **(B)** for FSK and **(C)** for F/I (bar graphs: average ± S.D. of 3 independent experiments).
